# Development, Statistical Optimization and Characterization of Fluvastatin Loaded Solid Lipid Nanoparticles: A 3^2^ Factorial Design Approach

**DOI:** 10.3390/pharmaceutics14030584

**Published:** 2022-03-08

**Authors:** Afzal Haq Asif, Prasanna Kumar Desu, Rajasekhar Reddy Alavala, Gudhanti Siva Naga Koteswara Rao, Nagaraja Sreeharsha, Girish Meravanige

**Affiliations:** 1 Department of Pharmacy Practice, College of Clinical Pharmacy, King Faisal University, Al-Ahsa 31982, Saudi Arabia; 2 College of Pharmacy, Koneru Lakshmaiah Education Foundation, Vaddeswaram 522502, India; prasanna.desu@gmail.com; 3 Shobhaben Pratapbhai Patel School of Pharmacy & Technology Management, SVKM’s NMIMS, V.L. Mehta Road, Vile Parle (W), Mumbai 400056, India; 4 Department of Pharmacy, School of Medical and Allied Sciences, Galgotias University, Greater Noida 203201, India; drgsnkrao@gmail.com; 5 Department of Pharmaceutical Sciences, College of Clinical Pharmacy, King Faisal University, Al-Ahsa 31982, Saudi Arabia; sharsha@kfu.edu.sa; 6 Department of Pharmaceutics, Vidya Siri College of Pharmacy, Off Sarjapura Road, Bangalore 560035, India; 7 Department of Biomedical Sciences, College of Medicine, King Faisal University, Al-Ahsa 31982, Saudi Arabia; gmeravanige@kfu.edu.sa

**Keywords:** fluvastatin sodium, 3^2^ factorial design, particle size, in vitro drug release, contour plots

## Abstract

The purpose of the present research work was to design, optimize, and evaluate fluvastatin-loaded solid lipid nanoparticles (FLV-SLNPs) using 3^2^ factorial design for enhancing the bioavailability. Fluvastatin has several disadvantages, including the low solubility and substantial first-pass metabolism resulting in a low (30%) bioavailability and a short elimination half-life. FLV-SLNPs were prepared using the nano-emulsion technique. For the optimization of the FLV-SLNPs, a total of nine formulations were prepared by varying two independent factors at three levels, using full factorial design. In this design, lipid (A) and surfactant (B) concentrations were chosen as independent factors, whereas entrapment efficiency (Y1) and in-vitro drug release (Y2) were selected as the dependent variables. Additionally, the prepared SLNPs were characterized for X-ray diffraction, Fourier transform-infrared spectroscopy, and differential scanning calorimetry. These studies revealed that there were no interactions between the drug and the selected excipients and the selected formulation components are compatible with the drug. Pharmacokinetic studies in rats confirmed significant improvement in AUC and MRT of SLNPs in comparison with the pure drug indicating the enhanced bioavailability of SLNPs. This study provides a proof-of-concept for the fact that SLNPs can be effectively developed via experimental factorial design, which requires relatively minimal experimentation.

## 1. Introduction

Fluvastatin (FLV), the first completely synthetic statin, is a drug of choice in hyperlipidemia as it effectively lowers the total and low-density lipoprotein cholesterol. The target for statin type of anti-hypercholesterolemic drugs is HMG-CoA reductase that catalyzes the conversion of HMG-CoA to mevalonate, the rate-limiting step in cholesterol biosynthesis. FLV is administered by oral route and has a half-life of about one to three hours. It gets hydrolyzed by cytochrome P3A in the intestine and liver [1]. Nevertheless, issues like the pH of the digestive tract, the length of time it takes to travel to the GI tract, and its poor aqueous solubility may also contribute to the impairment in the FLV distribution. Lymphatic uptake can be successfully used to improve the bioavailability of orally administered drugs using lipid-based delivery systems and is a less invasive approach compared to the parenteral route of administration. Often drugs first travel through the liver, but enhanced lymphatic delivery bypasses the hepatic first-pass and results in improved drug absorption, thus lymphatic channels flow straight through the thoracic system and thereby avoiding the portal flow [2]. In general, the primary role of the lymphatic system is to allow the lymph to move larger fats through the liver and allow the development of chylomicrons. Two methods are now proven to increase lymphatic uptake. These involve the inclusion of drugs into a lipophilic prodrug and drug lipophilicity enhancement [3]. It has been documented that the lipid-based delivery mechanisms were found to improve the bioavailability of lipid-soluble drugs by the lymphatic flow [4,5,6,7].

Researchers are also focusing on new ways to enhance the bioavailability of poorly aqueous soluble drugs, with or without changing their chemical structure like spray-drying [8,9], lyophilization [9], supercritical fluid extraction [10], co-crystal formation [11,12], microencapsulation, and particle size reduction, etc. [13,14]. However, on the whole, most of these methods have their pros and cons. Polymeric materials used in pharmaceuticals, biotechnology, food, and cosmetic applications, and consumer products can be defined as sustained and controlled release of active ingredients using polymers [15,16].

SLNPs are a novel type of drug carrier that enables controlled and targeted drug delivery via a variety of routes of administration. SLNPs’ size ranges between 1 to 1000 nanometers and are physiologically stable particles. SLNPs are often made from biodegradable and biocompatible materials such as phospholipids, fatty acids, mono, di, and triglycerides, all of which are naturally occurring in the human body [15]. However, as the particle size grows, the absorption becomes selective and undergoes slowly. It was seen in the experiment that polymeric nanoparticles get absorbed into the Microfold (M) cells of the intestines through the lymphatic system. This seems to be a common occurrence with the nano-sized materials which are well-suited to have been pulled into the lymphatic vessels by cells. The researchers observed that SLNPs greatly enhanced the bioavailability of the various poorly water-soluble drugs.

Accordingly, SLNPs have several advantages, including improved drug stability, suitability for both hydrophobic pharmaceuticals and lipophilic drugs, reducing the toxicity of drugs to specific tissues, the capacity to be produced in large quantities, and higher bioavailability. SLNPs have several disadvantages for various drugs, the most significant of which is a low loading efficiency due to polymeric transition during storage. The crystalline lipid matrix allows limited area for the drug molecule to bind leading to the encapsulation of hydrophobic and hydrophilic drugs.

The purpose of the work was aimed to formulate the FLV-SLNPs by high-speed homogenization and investigate the effect of independent parameters on % entrapment efficiency and in vitro drug release by applying the 3^2^ factorial design.

## 2. Materials and Methods

Fluvastatin was obtained as a gift sample from BMR Pharma and Chemicals, Hyderabad, Telangana, India. Glyceryl Monostearate and Kolliphor P188 were purchased from Pavani Chemicals, Hyderabad., India. Both chemicals and reagents used were of high purity.

The impact of numerous parameters like lipid: drug ratio, surfactant concentration, stirring speed, stirring time was investigated on encapsulation efficiency, particle size, and drug release as preliminary studies. The preliminary investigation led to the identification of independent variables and the determination of their minimal and maximal values. A trial version of the Design-of-Experiments (DOE) software (Version 13S, State-Ease, Inc., Minneapolis, MN, USA) was used to construct the 3^2^ factorial design. Table 1 lists the independent and dependent variables, along with their corresponding levels.

The SLNPs containing FLV were formulated using a hot homogenization approach and an ultra-sonication method. In a combination of methanol and chloroform, FLV and Glyceryl Monostearate were dissolved in the ratio of 1:1. The rotary flash evaporator was used to extract organic solvents from the sample. The embedded lipid layer was melted by heating the sample to a temperature of 5 °C above the melting point of the lipid. The hot aqueous phase was transferred to the heated oil phase and homogenized with a mechanical stirrer at 2500 rpm and 70 °C after dissolving the stabilizers in distilled water and heating it to the same temperature as the oil phase for 30 min. To get the required outcome, the coarse oil in water emulsion was sonicated for 25 min with a probe Sonicator. It was ultimately possible to obtain FLV-SLNPs by allowing the heated nanoemulsion to cool at ambient temperature before storing it at 4 °C in the refrigerator.

### 2.1. Factorial Design Approach

The maximum and minimum limits for independent variables were chosen based on the results of the preliminary investigation. The SLNPs were optimized by the use of a 3^2^ factorial design. Dependent variables such as Encapsulation efficiency was denoted as Y1, and in vitro drug release was denoted as Y2. The lipid content (A) and the surfactant concentration (B) were the independent variables. A conclusion is considered significant when the estimated *p*-value is less than 0.05. Using Design-Expert^®^ software 13.0 trial version, a desirability function was applied to all of the responses at the same time, optimizing them all concurrently. In the desirability function technique, the formulations were optimized by keeping A and B within the range of values employed in the current work, while Y1 and Y2 were kept at the lowest and highest possible levels, respectively. The feasible formulation compositions with a high desirability value for the specified goals were calculated. It was necessary to construct an overlay plot to determine the design space for desired responses. The suggested formulation was made and tested for a variety of reactions before being considered as an optimum formulation [11].

### 2.2. Particle Size Distribution and Zeta Potential

The Zetasizer (Nano ZS^®^, Malvern, UK) was used to determine the average particle size, size distribution, and the zeta potential of each formulation. In brief, freeze-dried powdered nanoparticle formulations were homogenized in Milli-Q water that had been sonicated for 30 min, filtered using a Millipore filter and a 0.22 membrane, and sterilized using the filtration [17].

### 2.3. Entrapment Efficiency (EE)

The prepared SLNPs dispersion was centrifuged at 15,000× *g* for 30 min at room temperature with a REMI cooling centrifuge to obtain the desired result. The free drug content of the supernatant is then determined by testing the supernatant [18]. The approach used the following equations to determine the EE.

% EE = (Amount of drug in SLNPs (mg) × 100)/(Amount of drug added (mg))(1)

### 2.4. In-Vitro Drug Release Studies

The dialysis bag technique has been used to calculate the release of FLV from SLNPs. A 2 mL of nanoparticulate dispersions (equivalent to 40 mg of FLV) was placed in a dialysis bag (molecular weight cut off, 12,000–14,000 Da), tied suitably on both sides, and placed into 100 mL of dissolution media. An open-ended bag was dipped in the pH 6.8 phosphate buffer, which was stirred at 100 rpm at 37 ± 0.2 °C. Fresh dissolving media was used at each time point to replace 2 mL of the previously withdrawn sample. After appropriate dilution, the amount of FLV in samples was measured using a UV spectrophotometer. Later in vitro drug release data was fit to different kinetic models to determine the mechanism of drug release.

### 2.5. FTIR Study

FTIR study was carried out to ensure the compatibility between pure drug and optimized formulation. The spectra was acquired using an FTIR spectrophotometer in the range 4000–500 cm^−1^ [19].

### 2.6. DSC Study

DSC analysis was executed for pure drug and optimized formulation. Around 4 mg of the sample was precisely weighed and was wrapped in standard aluminum pans with a lid by crimping. For each time the temperature ramping, the number of thermograms was reported between 30 and 300 °C by10 °C per minute. To do this, 50 mL of nitrogen gas was pumped into the reactor under a constant flow rate of 50 mL·min^−1^. These experiments were conducted to explore the physical properties of the pure drug and solid lipid nanoparticles [20].

### 2.7. X-RD Study

X-RD study was conducted using a Benchtop X-ray diffractometer. A glass sample holder was used to hold the sample. Cu K radiation was emitted at a current of 30 mA and a voltage of 40 kV. Samples were scanned from 10–80° with a step size of 0.05° [20].

### 2.8. Animal Studies

The in vivo experiments were conducted on Wistar rats (which weighed approximately 200 to 250 g each), in the Animal Research Centre of the Vishnu Institute of Pharmaceutical Education & Research, Hyderabad (VIPER/IAEC/2021/16, 22 March 2021). Food and water were provided in polypropylene enclosures for the animals. The animals’ proper care and feeding were given following the recommendations set out by the CPCSEA.

#### Pharmacokinetic Evaluation

The pharmacokinetic evaluation study was carried out by randomly dividing rats into two groups with six animals in each group. All the animals were maintained under standard conditions of temperature and humidity at 24 ± 2 °C and 60% RH. Animals were provided with water ad libitum and food intake was restricted for 18 h before the study. With the aid of a feeding cannula, the formulations were administered to the animals. Group, I was treated with pure fluvastatin suspension, while group II was treated with optimized formulation, FSLN9. Blood samples were collected from the tail vein at different intervals of 0, 0.5, 1, 2, 3, 4, 6, 12, 16, 20, and 24 h. The samples were transferred to heparinized tubes for further processing. The collected blood samples were processed by centrifugation at 3000× *g* for 15 min to separate the plasma which is stored at −20 °C for further analysis. RP-HPLC method was used to analyze the samples. Assessment of various pharmacokinetic parameters namely C_max_, T_max_, K_E_, T_1/2_, AUC (Area under the curve), AUMC (Area under moment curve), and MRT (Mean residence time) was done by using PK solver (a free tool available as an add-in for Microsoft excel) [21].

## 3. Results and Discussion

The results of the evaluation parameters of FLV-SLNPs were shown in Table 2. Both independent variables namely volume of lipid and concentration of surfactant were found to be showing a significant influence on the responses which was presented as a series of linear equations and response surfaces.

### 3.1. Particle Size and Zeta Potential

The particle size of all SLNPs formulations was found to be between 354.2 and 153.5 nm with a PDI of 0.148 to 0.212 (Table 2).

**Figure 1:**
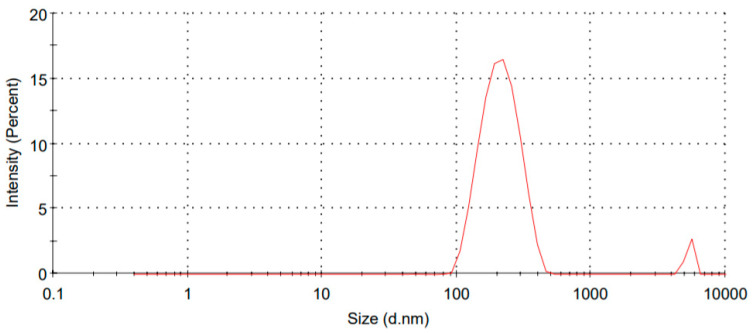
Size distribution study of formulation FSLN9.

The SLNPs were found to be distributed within the nanometer size range. The second peak in the size distribution graph may be due to a negligible number of particles having a coarse size or air bubble incorporated in the formulation during testing (Figure 1).

An increase in the concentration of both lipid and surfactant contributed to lowering the particle size of nanoparticles. In the range of −36.4 to −14.9 mV, the FLV-SLNP’s potential was discovered. Both formulations were determined to be stable based on the effects of Zeta potential. Table 2 shows the values of various evaluation parameters like particle size, PDI, zeta potential, and encapsulation efficiency.

### 3.2. Encapsulation Efficiency

The entrapment efficiency for FLV-SLNPs ranged from 58.62% to 80.46%, and as the concentration of lipid increased, the entrapment efficiency increased. The values were tabulated in Table 2.

### 3.3. In Vitro Drug Release Studies

**Figure 2:**
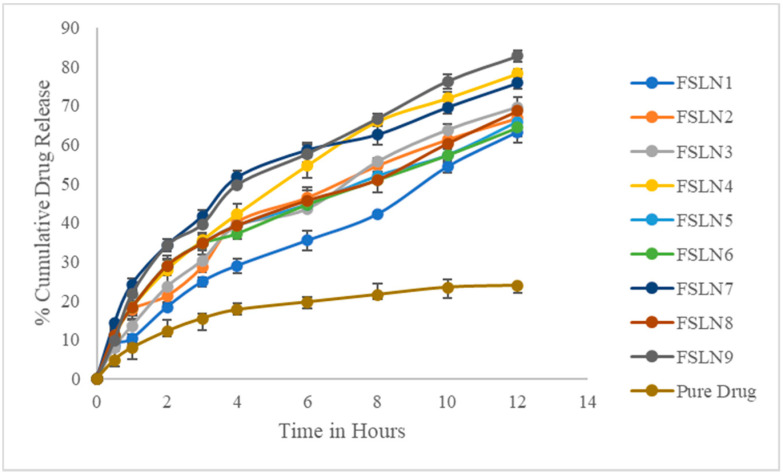
Cumulative percent drug release of all formulation (Error bars indicated standard deviation of 6 determinations).

In vitro, drug release studies were performed using a dialysis bag. The details about the total percentage drug release for each formulation are shown in Table 3, as well as in Figure 2. In this specific study, the researchers looked at the overall percentage of drug release within 12 h. On average, the percentage of drug release of FLSV-SLNPs varies between 63.24 ± 2.68 and 82.66 ± 1.48 at the end of 12 h. Among all the formulations, FSLN9 which contains a high concentration of lipid and surfactant showed 82.66 ± 1.48 of drug release at the end of 12 h. The release profile data were fitted into various kinetic models to understand the mechanism of release [22]. It was found that the release of drug from optimized formulation FSLN9 was found to follow zero-order release kinetics with non-Fickian diffusion mechanism.

### 3.4. Design Analysis

The mathematical relationship between variables was constructed using the Design-Expert 11.0 and then evaluated by checking its ability to estimate response variables and interacted to show that there are definite effects on responses.

The results observed for the nine formulations were analyzed and their formulations were compared to different models using the Design-Expert. It was observed that the best fit was a linear model. Table 4 shows the ANOVA values, as well as the values of R^2^, adjusted R^2^, predicted R^2^, SD, percent CV, and even the regression equation derived for each response. The R^2^, for all response variables, demonstrated a good fit of the model [23,24]. The F value for all response models was identified to be high which shows that the models were significant. *p*-value less than 0.05 showed that the model terms were statistically significant, demonstrating that the probability of error is less than zero. It has been observed that both A and B as independent variables.

#### 3.4.1. Effects on Encapsulation Efficiency (Y1)

The polynomial equation obtained from the study is given by (2)

Y1 (% EE) = +68.47 + 8.36A + 2.93B(2)

**Figure 3:**
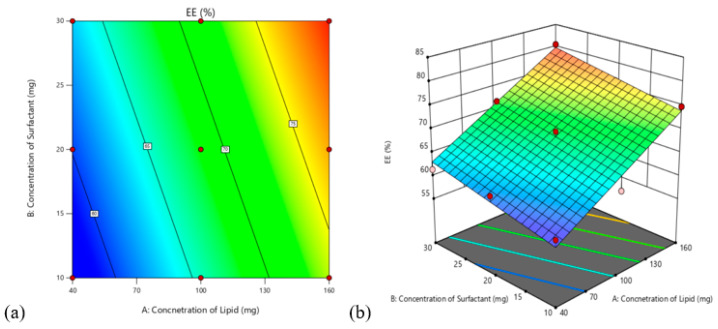
(**a**) 2D contour plot and (**b**) 3D response showing the effect of independent variables on response Y1.

The formula shown above is the quantitative expression of A and B (and only A and B) on the responses. A variable’s effect on the responses is represented by the magnitude of the coefficient, which displays all the values of the approximate coefficient on each response. Table 3 shows the coefficient and *p*-value for each component. F-Value of 73.64 means that the model is significant. With a high F-Value, there is just a 0.01% risk that may be attributed to noise. In this context, A and B were proven to be significant terms. A considerable influence on response Y1 was exerted by lipid concentration (*p* < 0.0001) and surfactant concentration, (*p* < 0.0027). A (*p* < 0.0001) and B (*p* < 0.0001) were significant influences on response Y2 (*p* = 0.0153). As shown in Figure 3, independent variables have a significant impact on response Y1.

#### 3.4.2. Effect on In-Vitro Drug Release Studies

The polynomial equation of In-vitro drug release studies obtained from the model is given by (3)

In vitro drug release studies (Y2) = +70.61 + 7.05A + 1.79B(3)

**Figure 4:**
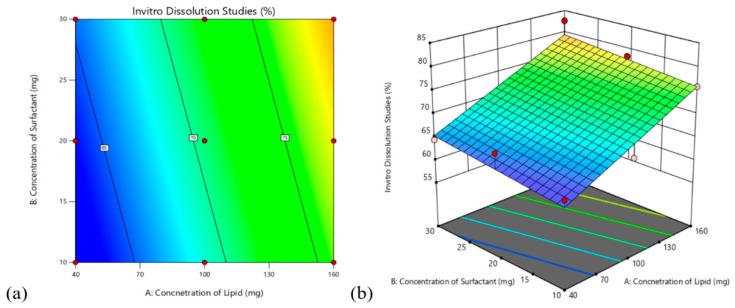
(**a**) 2D contour plot (**b**) 3D response showing the effect of independent variables on response Y2.

An increased drug release was observed with an increase in lipid and surfactant concentration of the formulation. Using the independent variables and their interaction principles, we can show the total impact on the parameters using the 2D counter and 3D response surface plots. Figure 4 shows both 2D and 3D plots showing the effect of independent variables on the response of % in vitro drug release.

#### 3.4.3. Validation of the Model

**Figure 5:**
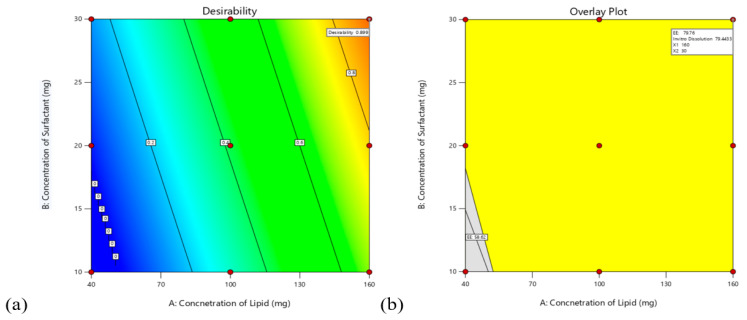
(**a**) Desirability plot by Numerical Optimization (**b**) Overlay plot by Graphical Optimization.

Using 3^2^ factorial designs, all nine formulations were subjected to experimental trials. Independent variables were optimized for all responses simultaneously using the desirability function after studying the effect of dependent and independent variables on responses. Y1 and Y2 responses have been transformed respectively into individual desirability scales d1 and d2 shown in Figure 5a,b. Both responses were set to be maximized. Based on the desirability value of 0.899, FSLN9 was selected as the optimized formulation. This factor level combination predicted the responses Y1 = 79.76%, Y2 = 79.44%. Three samples were eventually prepared to test optimal parameters and to measure the expected responses. The experimental values are shown to correlate closely with the predicted values, suggesting the performance of the 3^2^ factorial design resulted with the desired function for the evaluation and optimization of FVS-SLNP’s.

### 3.5. FTIR Study

**Figure 6:**
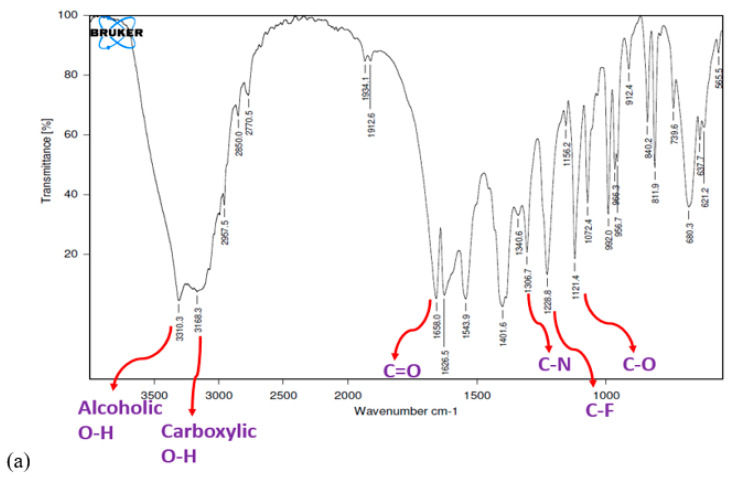

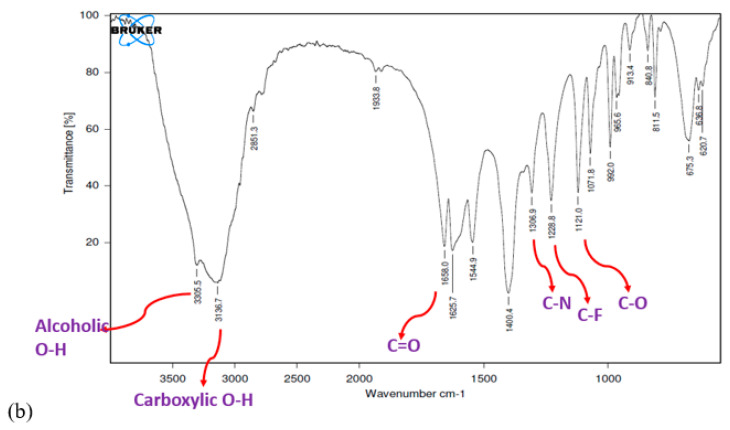
(**a**) Pure Fluvastatin (**b**) Optimized formulation FTIR Spectras.

Figure 6 shows the FTIR spectrum of pure fluvastatin and FLV-SLNPs. Characteristics peaks of the pure drug were compared with the peaks of the optimized formulation. The characteristic bands of pure FLV were identifiable. The optimized formulation showed no significant changes, indicating that the molecule is intact and has not reacted with the polymers.

### 3.6. DSC Study

**Figure 7:**
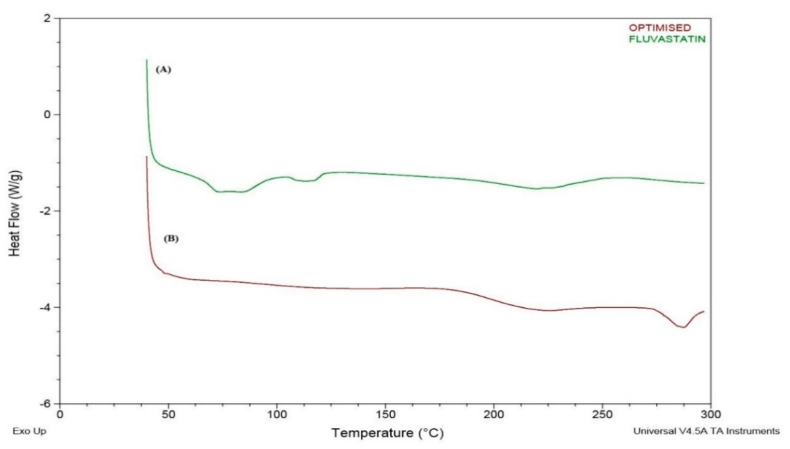
(**A**) Pure FLV, (**B**) optimized formulation thermograms.

Figure 7 shows the pure fluvastatin and its optimized formulation thermograms. It is observed that the endothermic peak representing the melting of fluvastatin in its pure thermogram is not appearing in the DSC thermogram of FLV-SLNPs indicating that the fluvastatin is not present in the crystalline form. This indicates that the drug is homogeneously dispersed with the lipid matrix without recrystallization. The same was confirmed through XRD studies.

### 3.7. X-RD Study

**Figure 8:**
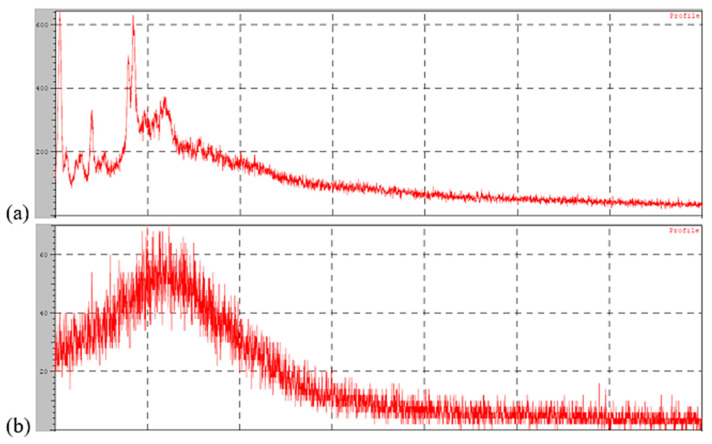
X-ray Diffraction Pattern of (**a**) pure FLV (**b**) optimized formulation.

The diffraction pattern of pure fluvastatin showed characteristic high energy peaks, indicating the crystalline structure shown in Figure 8a. However, the developed formulation did not show the same high energy peaks (Figure 8b). A significant reduction in peak intensities indicates the loss of crystallinity of fluvastatin in SLNPs [25]. This may be due to the presence of excipients and also due to solubilization of the active in the lipidic carrier.

### 3.8. Pharmacokinetic Evaluation

**Figure 9:**
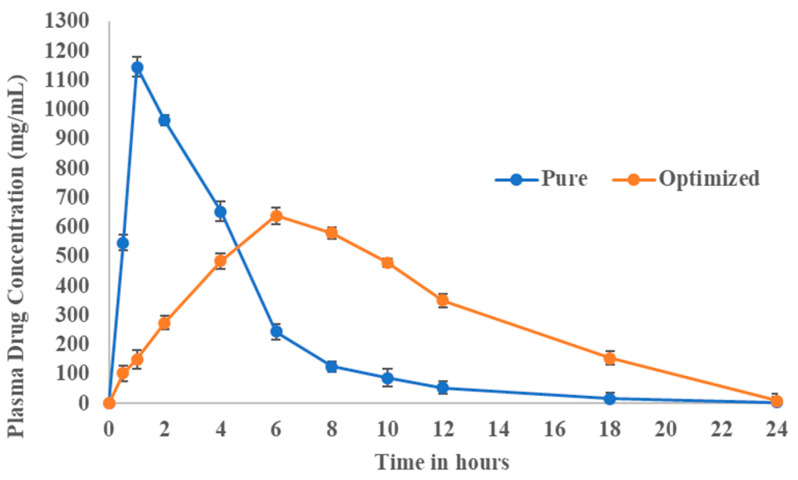
Pure fluvastatin and its optimized formulation plasma time profiles (Data represents mean of 6 determinations ± SEM).

The plasma concentration vs. time profile of fluvastatin in rat plasma, as shown in Figure 9, revealed that pure drug displayed maximum drug concentration at 1 h and after that rapid declined in plasma drug concentration (Table 5). After 8 h negligible amount of drug was found in plasma indicating faster elimination after rapid absorption. Whereas, in the case of optimized formulation, the concentration of drug in plasma was gradually increased up to 6hr after that concentration was slightly decreased up to 24 h. Pharmacokinetic data revealed that the developed formulation showed drug release slowly and for a prolonged period that lasted up to 24 h. Although there was a reduction in C_max_, the overall bioavailability of the developed nanoparticulate formulation was enhanced as observed from the increased AUC values. AUC and AUMC were calculated using the trapezoidal rule and were further used to calculate MRT [26]. Various pharmacokinetic parameters such as AUC, AUMC, and MRT of pure drug and developed formulation were also determined and results were shown in Table 5. The mean residence time of the drug was more for the optimized fluvastatin formulation indicating an extended duration of action compared to that of pure drug.

## 4. Conclusions

The investigation revealed that FLV-SLNPs prepared by glyceryl monostearate as lipid carrier and Kolliphor P188 as surfactant by high-speed homogenization method using 3^2^ factorial design. Through the use of Design-of-Experiments, independent variables can be selected. The amount of lipid and surfactant concentrations influenced the encapsulation process and in vitro drug release. The resulting SLNPs have a smooth surface and are in the 600–700 nm size range. In the FTIR and DSC studies, no drug-lipid interaction was found. X-RD study discloses the pure drug and optimized formulation in crystalline nature. Based on results it is concluded that the hot homogenization process for FLV-SLNPs is a feasible option for controllable release of fluvastatin and factorial design can be successfully employed for achieving the desirable characteristics of the formulation.

## Figures and Tables

**Table 1:** Design layout of all formulations by 3^2^ factorial design.

Formulation Code	Variables in Coded Form				Response	
	Amount of Lipid (A)		Amount of Surfactant (B)			
	Coded Values	Actual Values (mg)	Coded Values	Actual Values (mg)	Encapsulation Efficiency (Y1)	In Vitro Drug Release (Y2)
FSLN1	−1	40	−1	10	58.62	63.24
FSLN2	−1	40	0	20	61.32	66.84
FSLN3	0	100	0	20	69.54	69.65
FSLN4	1	160	0	20	76.2	78.28
FSLN5	0	100	−1	10	62.38	65.9
FSLN6	−1	40	1	30	61.38	64.44
FSLN7	1	160	−1	10	74.82	75.86
FSLN8	0	100	1	30	71.54	68.62
FSLN9	1	160	1	30	80.46	82.66

**Table 2:** Evaluation parameters of FLV-SLNP’s.

Formulation Code	Particle Size (nm)	PDI	Zeta Potential (mV)	Encapsulation Efficiency (%)
FSLN1	354.2	0.152	−36.4	58.62
FSLN2	324.8	0.168	−32.2	61.32
FSLN3	298.6	0.186	−28.8	69.54
FSLN4	265.8	0.182	−26.6	76.2
FSLN5	248.2	0.176	−24.2	62.38
FSLN6	243.8	0.168	−21.5	61.38
FSLN7	214.2	0.212	−20.4	74.82
FSLN8	189.5	0.168	−18.6	71.54
FSLN9	153.5	0.148	−14.9	80.46

**Table 3:** In-vitro drug release data of all formulations (Data represents mean of six determinations *±* standard deviation).

Time	FSLN1	FSLN2	FSLN3	FSLN4	FSLN5	FSLN6	FSLN7	FSLN8	FSLN9	Pure Drug
0.5	8.54 ± 1.54	10.65 ± 2.54	7.96 ± 1.12	10.86 ± 1.42	11.2 ± 1.58	11.2 ± 1.42	14.25 ± 0.68	11.2 ± 1.14	9.68 ± 1.54	4.8 ± 1.24
1	10.24 ± 1.24	17.65 ± 0.88	13.54 ± 2.54	18.24 ± 3.14	18.24 ± 2.98	18.24 ± 1.24	24.25 ± 1.37	18.24 ± 1.62	21.65 ± 1.84	7.9 ± 2.32
2	18.34 ± 0.84	21.24 ± 1.54	23.54 ± 3.24	27.96 ± 1.28	28.97 ± 1.46	28.97 ± 2.54	34.25 ± 1.68	28.97 ± 1.87	34.12 ± 0.24	12.18 ± 1.48
3	24.85 ± 1.24	28.63 ± 1.24	30.32 ± 2.48	35.42 ± 1.47	34.65 ± 2.88	34.65 ± 1.36	41.68 ± 1.47	34.65 ± 1.25	39.48 ± 0.86	15.42 ± 2.82
4	28.96 ± 1.86	40.19 ± 1.88	39.18 ± 1.84	42.17 ± 2.57	39.25 ± 1.34	37.25 ± 1.47	51.68 ± 1.62	39.25 ± 1.93	49.68 ± 0.14	17.64 ± 1.32
6	35.42 ± 2.45	46.45 ± 2.62	43.54 ± 1.24	54.68 ± 3.24	44.69 ± 1.58	44.69 ± 0.96	58.66 ± 187	45.69 ± 2.57	57.65 ± 2.24	19.64 ± 1.52
8	42.17 ± 0.54	54.65 ± 0.62	55.65 ± 0.98	65.98 ± 1.26	51.98 ± 1.21	50.98 ± 0.85	62.64 ± 2.68	50.98 ± 3.14	66.65 ± 1.36	21.54 ± 1.26
10	54.35 ± 1.68	61.35 ± 1.74	63.79 ± 1.42	71.94 ± 1.57	57.32 ± 2.76	57.32 ± 1.87	69.65 ± 1.58	59.32 ± 1.52	76.24 ± 1.87	23.42 ± 2.74
12	63.24 ± 2.68	66.84 ± 2.14	69.65 ± 2.66	78.28 ± 0.98	65.9 ± 1.92	64.44 ± 2.24	75.86 ± 1.58	68.62 ± 1.24	82.66 ± 1.48	23.87 ± 1.98

**Table 4:** ANOVA data for all responses.

	Value	F-Value	*p*-Value
(A) Encapsulation Efficiency			
Model	Linear		
R^2^	0.9609	73.64	<0.0001
Adj iR^2^	0.9478		
Pred iR^2^	0.9099		
Adeq Precision	21.8699		
(B) In vitro drug release studies			
Model	Linear		
R^2^	0.8681	19.75	<0.0023
Adj iR^2^	0.8242		
Pred iR^2^	0.7006		
Adeq Precision	10.79		

**Table 5:** Pharmacokinetic data of pure fluvastatin and its optimized formulation.

Pharmacokinetic Data	Pure Fluvastatin	Optimized Formulation
C_max_ (ng/mL)	1146	640
t_max_ (h)	1	6
T_1/2_(h)	2.9	2.9
AUC_(0–24)_ (mg/mL.h)	5122.5	7298.2
AUC_(0–∞)_ (mg/mL.h)	5135.2	7336.24
AUMC_(0–∞)_ (mg/mL.h^2^)	20,696.90	66,578.74
MRT (h)	4.03	9.07
K_E_ (h^−1^)	0.43	0.49

## Data Availability

Not applicable.
